# Bacterial Alterations in Post-Cholecystectomy Patients Are Associated With Colorectal Cancer

**DOI:** 10.3389/fonc.2020.01418

**Published:** 2020-08-12

**Authors:** Xinhua Ren, Jun Xu, Yuanyuan Zhang, Guodong Chen, Yiwen Zhang, Qing Huang, Yulan Liu

**Affiliations:** ^1^Department of Gastroenterology, Peking University People's Hospital, Beijing, China; ^2^Clinical Center of Immune-Mediated Digestive Diseases, Peking University People's Hospital, Beijing, China; ^3^Institute of Clinical Molecular Biology & Central Laboratory, Peking University People's Hospital, Beijing, China

**Keywords:** cholecystectomy, the duration after cholecystectomy, 16S rRNA gene sequencing, bacterial alterations, colorectal cancer

## Abstract

**Background:** Although increasing evidences showed a correlation between cholecystectomy and the prevalence rate of colorectal cancer (CRC), and shed light on gut microbiota in colorectal pathogenesis, only a few studies focused on microbial alterations after cholecystectomy, and its sequent role in carcinogenesis and progression of CRC has not been reported. Thus, we aimed to investigate the bacterial alterations and tried to clarify their clinical significance.

**Methods:** 104 subjects were enrolled and divided into post-cholecystectomy patients (PC, *n* = 52) and healthy controls (HC, *n* = 52). To investigate the bacterial role in carcinogenesis, PC patients were further separated into preCA_CRC (patients with precancerous lesions and/or CRC, *n* = 9) and non-CA (patients without precancerous lesions and CRC, *n* = 43) based on the histopathology. Qualified stool samples were collected for 16S rRNA gene sequencing to analyze the bacterial profile.

**Results:** Our data showed noteworthy compositional and abundant alterations of bacterial microbiota in PC patients, characterized as *Bacteroides ovatus, Prevotella copri*, and *Fusobacterium varium* remarkably increased; *Faecalibacterium prausnitzii, Roseburia faecis*, and *Bifidobacterium adolescentis* significantly decreased. Additionally, the duration after cholecystectomy was the critical factor that affected bacterial composition. Machine learning-based analysis showed a pivotal role of *Megamonas funiformis* in discriminating PC from HC subjects and involving in the progression of CRC.

**Conclusions:** The bacterial dysbiosis may associate with CRC in PC patients, and the duration after cholecystectomy was highlighted as an important factor. Altered bacterial microbiota was likely to play a pivotal role in related-disease in the long-term follow-up of PC patients.

## Introduction

With the changes of the modern lifestyle, the prevalence of cholelithiasis has steadily increased (1). Cholecystectomy is the gold standard for the treatment of symptomatic cholelithiasis (2), and the number of this surgery has also increased significantly. According to the information statistics, there are about 800,000 cases of cholecystectomy in the United States each year, and the number is also increasing in China annually (3, 4).

Cholecystectomy was considered nearly harmless in the past. However, growing evidences have shown that the prevalence of post-cholecystectomy syndrome (such as abdominal distention and abdominal pain) has increased in recent years, up to 10–47% (5). Meanwhile, it was reported that diabetic patients after cholecystectomy showed a slight deterioration in postprandial glycemic control in a clinical trial (6). Two large population-based studies suggested that cholecystectomy was likely to be one of the independent risk factors for non-alcoholic fatty liver disease (NAFLD) (7, 8). It was inferred that cholecystectomy might increase the risk of metabolic syndrome. In addition, some meta-analyses suggested that cholecystectomy probably raised the prevalence rate of colorectal cancer (CRC), particularly in the risk of right colon cancer (9–12).

Previous evidence showed that gut microbiome played crucial roles in colorectal carcinogenesis (13). Otherwise, a similar effect has not been reported in post-cholecystectomy (PC) patients complicated with CRC yet. There was a complex crosstalk between BAs and gut microbiome, BAs can modulate gut microbial composition (14–16), and BAs are stored in the gallbladder and metabolized by the gut microbiota. Therefore, we speculated that cholecystectomy likely had a huge impact on intestinal microbial homeostasis and facilitated CRC carcinogenesis and progression.

During the past decade, there were a few studies about gut microbiota after cholecystectomy. Keren et al. reported that cholecystectomy significantly affected the bacterial composition of cholelithiasis patients, with a remarkable increase in the abundance of *Bacteroidetes* (17). A study in China found that gut bacterial composition in healthy people changed with age, but the change was lost in PC patients, and the abundance of *Bacteroidetes* decreased in PC patients (18). Additionally, another study which contained 27 PC patients showed a subtle difference in the diversity of gut microbiota between the cholecystectomy and control groups (19). Nevertheless, these previous results were controversial, and the characterization in gut microbiota after cholecystectomy is still unclear. In addition, these studies analyzed at the genus level, ignored environmental factors and related diseases, and the character of bacterial alterations has not been further studied. Therefore, we intended to explore the alterations and roles of bacterial microbiota after cholecystectomy, and tried to clarify the clinical significance of the alterations.

## Materials and Methods

### Study Design and Sample Collection

Because there were few studies on the bacterial microbiota after cholecystectomy, and some existing studies had contradictory results on the abundance changes of the same genus of bacteria. In the early stage, we performed a pretest study for sample size evaluation, and considered the 10% loss of follow-up rate, therefore *n* was equal to 52 in each group.

Then a total of 52 PC patients were enrolled after cholecystectomy above 6 months and <25 years in Peking University People's Hospital from January 2018 to October 2018. Meanwhile, 52 healthy controls (HC) without any biliary diseases, tumors and traumatic ruptures were selected from health physical examination volunteers to match with PC patients. All involved subjects underwent colonoscopy within 6 months and were asked to avoid using probiotics and antibiotics at least 2 weeks before sampling. The demographic and basic clinical data of each group were recorded. Additionally, 9 of the PC patients had precancerous lesions and/or CRC, which confirmed by colonoscopy mucosal pathology. Firstly, we performed a 1:1 matched case-control group analysis. Subsequently, according to the absence and presence of precancerous lesions and/or CRC, we divided the PC patients into non-CA and preCA_CRC for subgroup analysis.

Fecal samples were collected in a Stool Collection Tube with Stool Stabilizer (*German, Stratec Molecular*) and then were separated and stored with ultra-hypothermia liquid nitrogen until microbial analysis.

### DNA Extraction, 16S rRNA Gene Amplification and Sequencing

The total microbial genomic DNA was extracted from stool samples using the PSP® Spin Stool DNA Kit (*German, Stratec Molecular*). PCR amplification of 16S rRNA genes used barcoded primers specific to the V3–V4 variable region (357F, 806R) (20). Each PCR product was purified and amplified again to link with sample-specific barcodes. Sequencing was performed using the instrument secondary analysis of MiSeq Reporter software (MSR).

### 16S rDNA Sequence Analysis

The main software used for sequence analysis were Vsearch v2.8.1 (21) and Usearch v10 (bit 32) (22). The original data were merged by double-ended sequences by Vsearch, followed by data quality control, excision of primers and barcode, and the 164,772 sequence was removed, leaving 20,594,067 sequences. Then redundant sequences and sequences with <100 occurrences were removed by Vsearch. A total of 12,949,719 redundant sequences were removed and 9,012 high-quality sequences were obtained.

Exact sequence variants (ESVs) method was performed to filter chimeras (23), and 3,387 high-quality amplicons were obtained. Operational taxonomic units (OTUs) were aligned using the Vsearch and taxonomically classified using the reference sequence rdp_16s_v16_sp.fa. All specimens were sampled into the same amount of reads through Usearch V10, resulting in a total of 3,113,022 Reads and 3,179 OTUs. Among them, 0 OTUs appeared in all samples, 52 OTUs appeared in 90% of samples, and 1,637 OTUs appeared in 50% of samples.

### Statistical Analysis and Data Visualization

R 3.4.1 software with the *ggplot2* package was used for visualization. The categorical variables were described by the number of cases, using the chi-square test or Fisher's exact test. The continuous variables were described by the mean ± standard deviation (Mean ± SD). The Mann-Whitney U non-parametric test and Kruskal-Wallis H non-parametric test was used for comparison. Correlation analysis was performed using Spearman's test; the *p*-value was corrected with a false discovery rate (FDR) and only a significant correlation was visualized with the *pheatmap* package. And *p* ≤ 0.05 was considered to be statistically significant.

## Results

### Characterization of Enrolled Subjects

A total of 104 subjects enrolled in our study, and their characterizations were showed in Table 1. PC patients, who suffered from acute and chronic cholecystitis, cholelithiasis, gallbladder polyps, and traumatic gallbladder rupture, were recruited after cholecystectomy at a mean duration of 9.48 ± 8.02 years. No significant differences were found between the two groups in terms of gender, age, and BMI. In addition, we compared complications ratios, for example, NAFLD, HBP, T2DM, etc., and there were no remarkable differences in the two groups. So did in laboratory indexes (for example TBA, ALT, AST, etc.). The above data indicated that there were no markedly alterations of clinical characteristics in PC patients, which were in accordance with the previous studies (24).

**Table 1:** Demographic and clinical profiles of post-cholecystectomy patients and healthy controls.

	**Health control**	**Post-cholecystectomy**	***p*-value**
	**(*n* = 52)**	**(*n* = 52)**	
Gender (M/F)	18/34	18/34	1.000
Age	59.71 (±11.95)	60.02 (±11.53)	0.735
BMI	24.38 (±3.63)	25.71 (±3.47)	0.787
CRC family history	1	3	0.308
**Complication**			
NAFLD	16 (30.8%)	22 (42.3%)	0.22
HBP	27 (51.9%)	27 (51.9%)	1.000
T2DM	15 (28.8%)	20 (38.5%)	0.299
HLP	10 (19.2%)	15 (28.8%)	0.326
CHD	3 (5.8%)	6 (11.5%)	0.244
**Laboratory tests**			
WBC (× 10^9^/L)	6.10 (±1.59)	6.05 (±1.47)	0.388
NE (%)	57.77 (±8.13)	56.00 (±8.87)	0.288
HB (g/L)	132.82 (±12.76)	137.83 (±14.02)	0.495
PLT (× 10^9^/L)	232.22 (±53.60)	221.37 (±66.46)	0.392
ALT (U/L)	18.63 (±8.47)	21.37 (±9.34)	0.587
AST (U/L)	19.88 (±5.67)	21.87 (±7.27)	0.883
GGT (U/L)	29.02 (±22.53)	25.96 (±17.13)	0.285
ALP (U/L)	77.55 (±18.78)	85.32 (±32.18)	0.079
TBA (μmol/L)	3.2 (±0.58)	2.83 (±0.65)	0.846
ALB (g/L)	39.44 (±3.72)	41.90 (±4.87)	0.146

### Cholecystectomy Altered Community Diversity of Bacterial Microbiota

**Figure 1:**
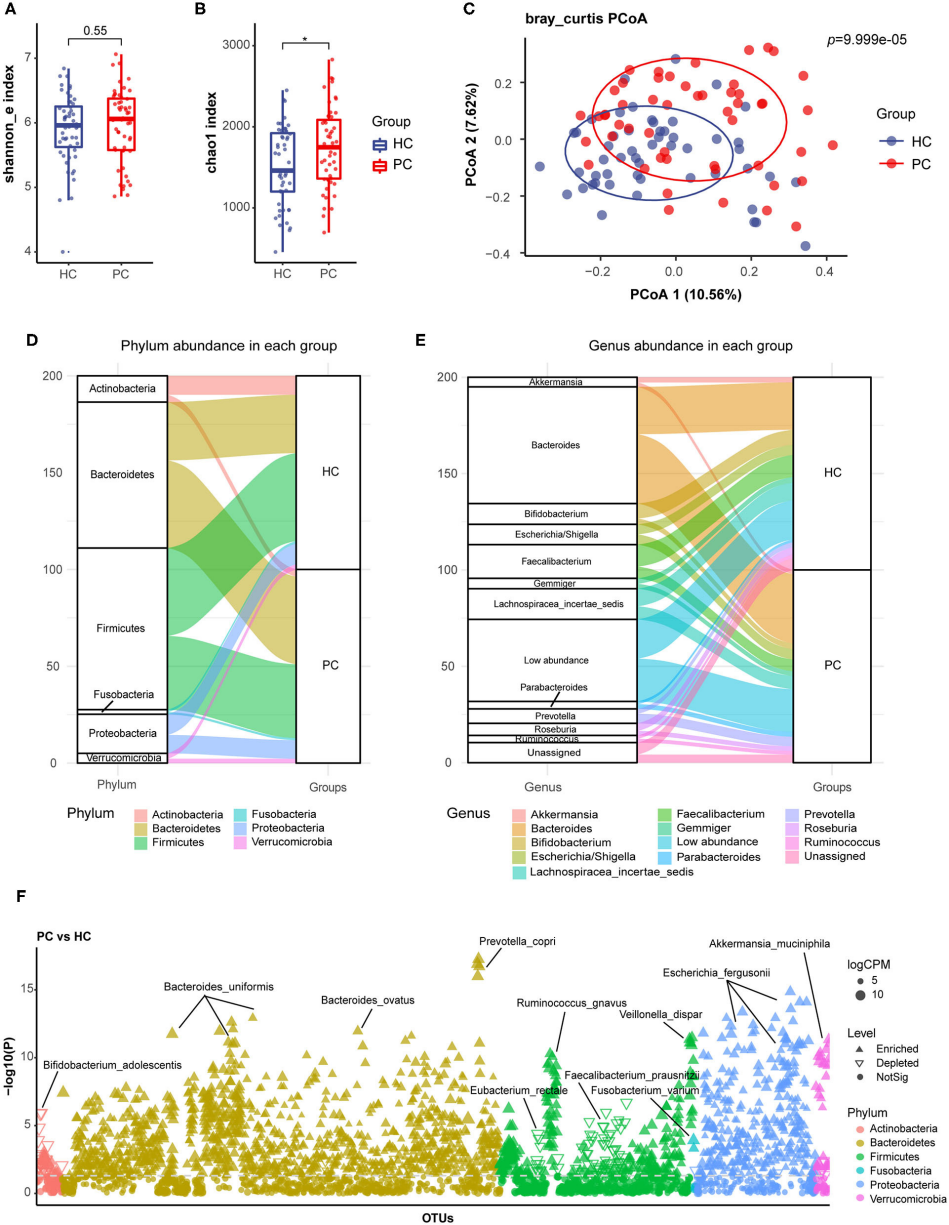
Alterations of fecal bacterial microbiota profile in PC patients. The alpha diversity of PC and HC group, **(A)**, Shannon index; **(B)** Chao1 index. *p < 0.05. **(C)** Principal coordinate analysis (PCoA) of bacterial beta-diversity based on Bray Curtis distance. The Sankey diagram to visualize the bacterial composition at the phylum **(D)** and genus **(E)** level. **(F)** Bacterial species with abundance differentiation in PC group compared with HC group in the Manhattan diagram. Differences between two groups were shown as point shape indicated OTU enriched, depleted or not significant; point size indicated the abundance of OTU. FC, fold change; CPM, counts per million; HC, healthy controls; PC, post-cholecystectomy.

Firstly, we compared the alpha diversity of the two groups by Shannon index (Figure 1A) and Chao1 index (Figure 1B), which represented species diversity and richness, respectively. In our study, there was a higher chao1 index and a similar Shannon index in PC patients. Then, principal coordinate analysis (PCoA) of beta diversity showed separated clusterings in PC and HC groups (Adonis test, *p* = 9.999e-05, Figure 1C). These data indicated that the species richness of bacterial microbiota increased, and the composition quite altered in PC patients.

We further analyzed the bacterial composition in PC and HC groups, and delineated compositional structure which was mainly constituted by *Bacteroidete*s and *Firmicutes* at the phylum level (Figure 1D); *Bacteroides, Bifidobacterium, Escherichia*, and *Faecalibacterium* at the genus level (Figure 1E) of two groups. Additionally, the relative abundance of *Bacteroides, Parabacteroides*, and *Prevotella* increased; and the relative abundance of *Faecalibacterium* and *Bifidobacterium* decreased in PC patients at the genus level.

### Bacterial Species With Abundance Differentiation in PC Patients vs. HC Subjects

By analysis and identification bacterial species with abundance differentiation in each group, we found that the abundance of *Bacteroides ovatus (B. ovatus), Parabacteroides distasonis (P. distasonis), Prevotella copri (P. copri)*, and *Fusobacterium varium (F. varium)* remarkably increased; additionally, a significant reduction in the abundance of *Faecalibacterium prausnitzii (F. prausnitzii), Roseburia faecis (R. faecis), Eubacterium rectale (E. rectale)*, and *Bifidobacterium adolescentis (B. adolescentis)* was observed in PC patients compared with HC subjects (Figure 1F).

*B. ovatus* and *P. distasonis* were reported to express bile salt hydrolase (BSH) and take part in bile acid metabolism (25–27). *P. copri* and *F. varium* were proved to promote inflammation (28, 29). However, *F. prausnitzii* and *R. faecis* were confirmed to participate in short-chain fatty acids (SCFAs) biosynthesis. *B. adolescentis* could secrete small molecule anti-inflammatory substances and inhibit inflammation (30). *Eubacterium rectale* could degrade starch in the human intestine and competitively inhibited the growth of harmful bacteria (31).

Based on these results, we inferred that fecal bacterial microbiota underwent a remarkable alteration after cholecystectomy, which was characterized as the accumulation of species with pro-inflammatory effects involved in bile acid metabolism, and the depletion of protective species with anti-inflammatory effects or producing SCFAs.

### Group-Specific Microbiota in PC Patients Differentiated Disease Status

**Figure 2:**
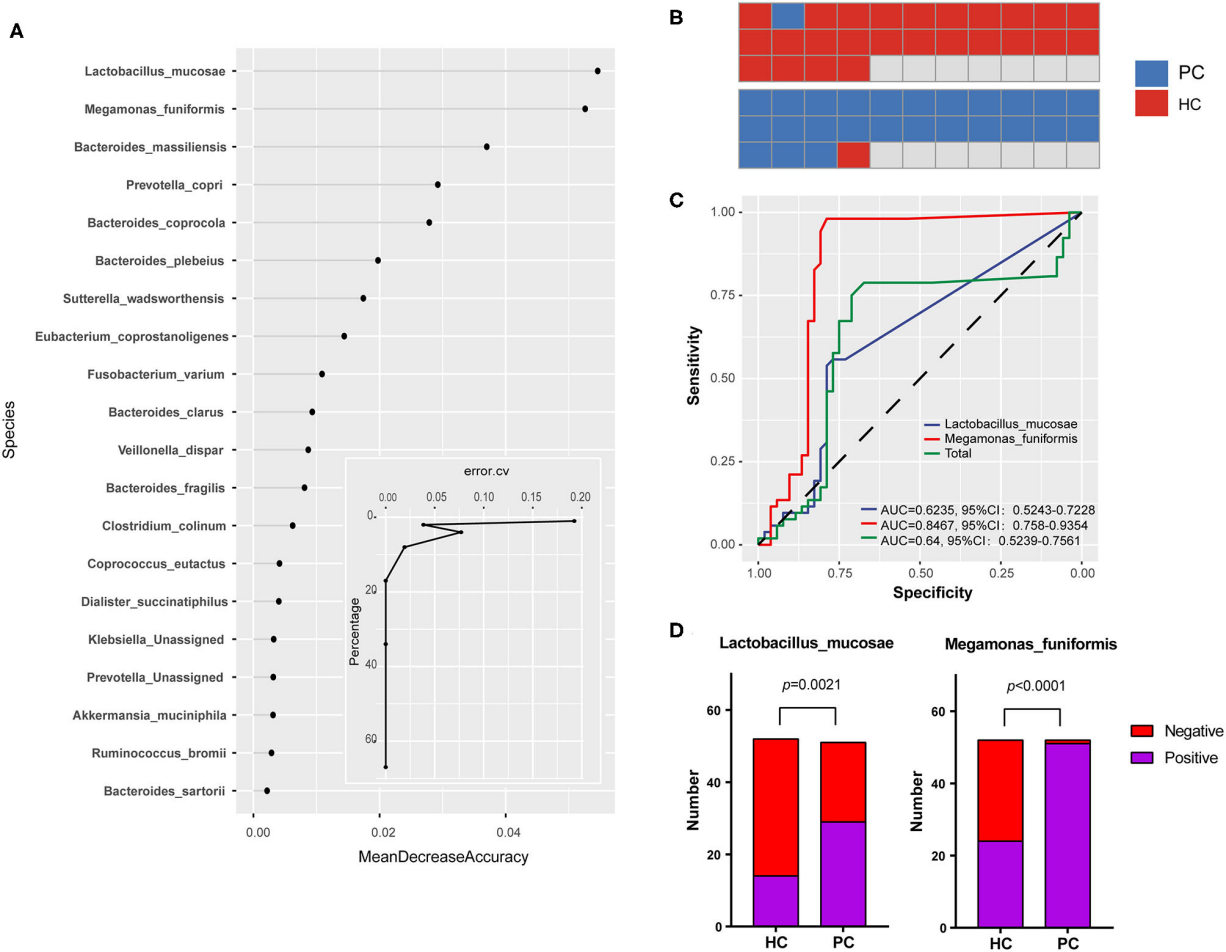
Group-specific microbiota in PC patients. **(A)** Bacterial contribution values to the disease classification at the species level and the curve of prediction error rate. Random Forest package was used for comparative analysis and took random seeds as 315. **(B)** The prediction accuracy rate of biomarkers in the validation step. **(C)** Receiver operating characteristic (ROC) curve for biomarkers in the validation step. **(D)** The comparison for positive test rates of biomarkers in PC and HC groups, respectively. HC, healthy controls; PC, post-cholecystectomy.

We next performed a machine learning analysis to confirm the group-specific microbiota, randomly selected half of the samples from each group to make up the model in the exploration step, and screened out the 20 most important species which contributed a lot to the disease classification to visualize (Figure 2A). According to the curve in Figure 2A, the prediction error rate going down to 4% when the top two species were enrolled in. Therefore, *Megamonas funiformis (M. funiformis)*, and *Lactobacillus mucosae (L. mucosae)* contributed largely to the sample classification, and were selected as biomarkers of PC patients. Then, using biomarkers for further validated in the remaining subjects of each group, the prediction accuracy rate was 96.2% (Figure 2B). Taking the two biomarkers into the validation step (Figure 2C), the area under the curve (AUC) of *L. mucosae* was 0.62, and the AUC of *M. funiformis* was 0.85. Combined with the positive test rate in two groups, respectively (Figure 2D), we verified that *M. funiformis* and *L. mucosae* were characteristic species which could differentiate PC patients from HC subjects.

*L. mucosae* was a protective strain colonized on mucosa and also involved in bile acid metabolism (32–36). *M. funiformis* was classified into the genus *Megamonas* of the family *Verimellaceae* in the phylum of *Firmicutes* (37), and its function is still unclear at present. In addition, as PC biomarkers, the alterations and more characters of the two species need further studies.

### Correlations Between Environmental Factors and Bacterial Composition

**Figure 3:**
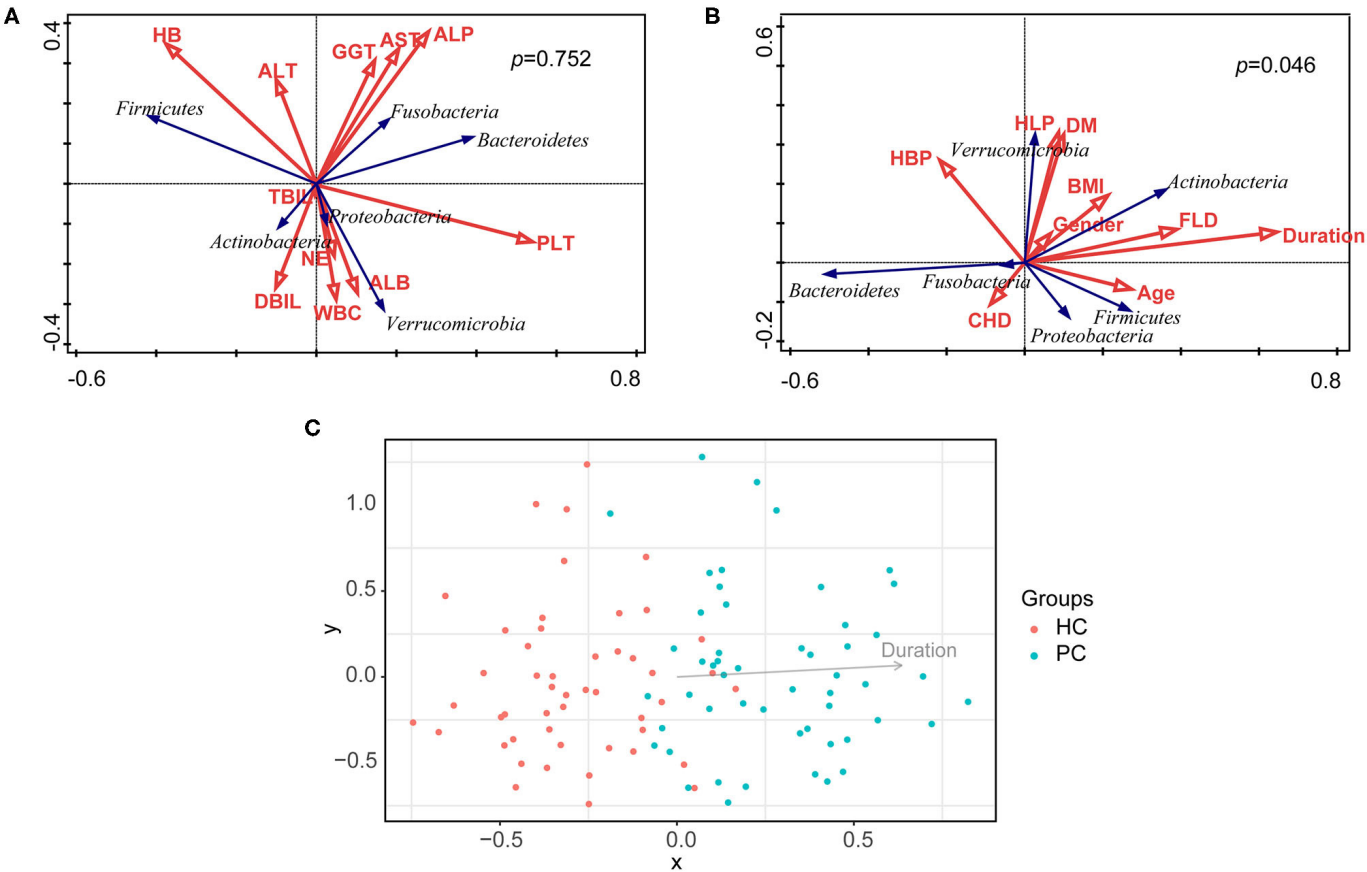
Correlation analysis of clinical characteristics, environmental factors, and bacterial composition. **(A)** Correlation analysis of clinical characteristics and bacterial composition of all samples. **(B)** Correlation analysis of environmental factors and bacterial composition of all samples. **(C)** Correlation analysis of the duration after cholecystectomy and bacterial composition of all samples. HC, healthy controls; PC, post-cholecystectomy.

Taking nine clinical characteristics (such as TBA and ALT) and 10 environmental factors (such as BMI and the duration after cholecystectomy) into account, no correlation was observed between the model of overall clinical characteristics and the bacterial composition of all samples (*p* = 0.075, Figure 3A); whereas the model of overall environmental factors was significantly correlated with the bacterial composition (*p* = 0.046, Figure 3B). Furthermore, we examined every environmental factor and found that only the duration after cholecystectomy demonstrated the most pronounced correlation with bacterial composition (*p* = 0.002, Figure 3C). With the increase of the duration, bacterial dysbiosis after cholecystectomy was more obvious. These findings suggested that the long-term follow-up of PC patients should attract more attention.

### Community Diversity and Bacterial Species With Abundance Differentiation in preCA_CRC vs. Non-CA Patients

**Figure 4:**
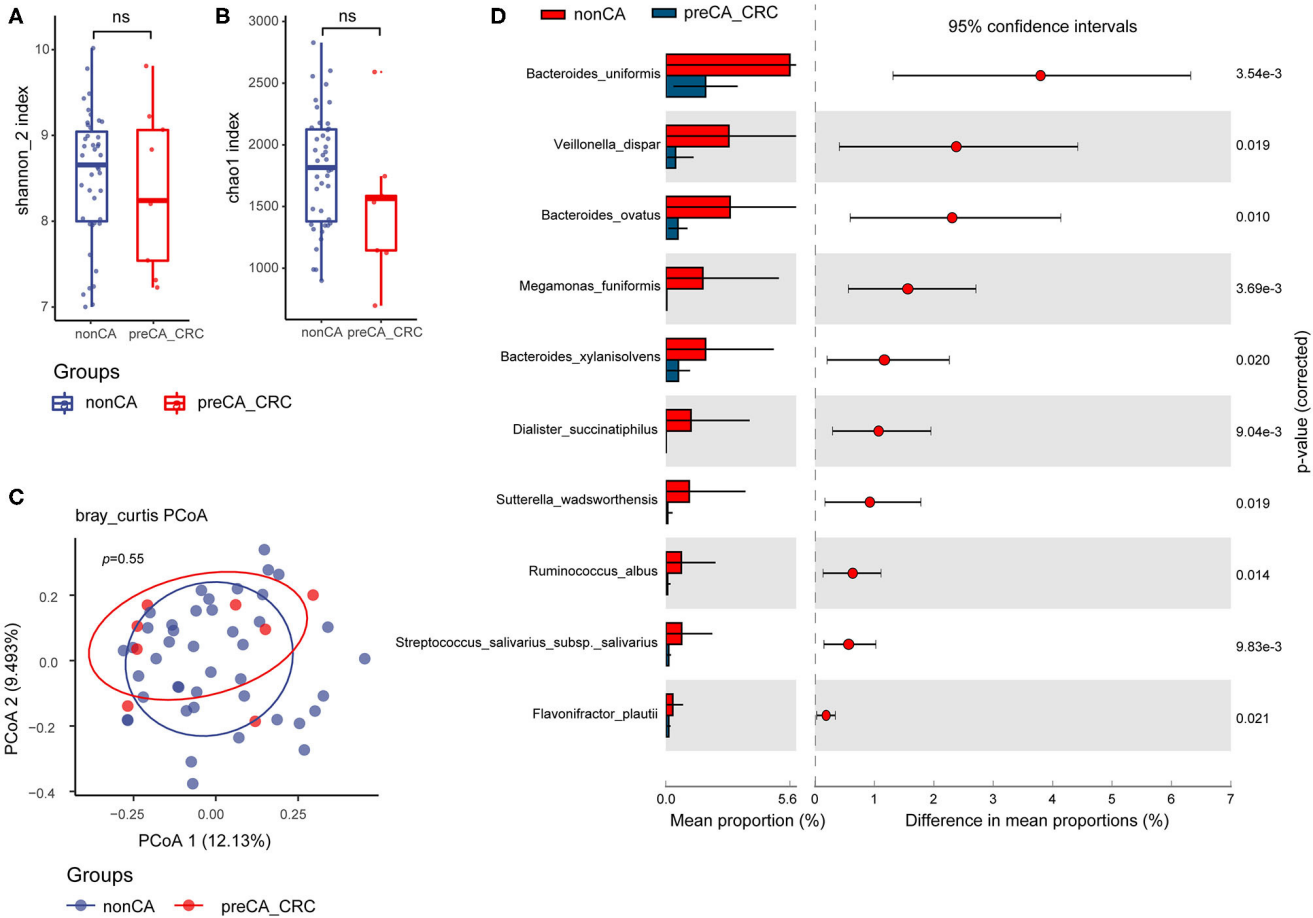
Compared with non-CA patients, alteration of fecal bacterial microbiota profile in preCA_CRC patients. The alpha diversity of preCA_CRC and non-CA patients, **(A)** Shannon index; **(B)** Chao1 index. ns, not significant. **(C)** PCoA of bacterial beta-diversity based on Bray Curtis distance. **(D)** Bacterial species with abundance differentiation in preCA_CRC patients compared with non-CA patients by STAMP. preCA_CRC, PC patients with precancerous lesions and/or CRC; non-CA, non-cancerous PC patients.

Similarly, we compared the alpha diversity indexes of preCA_CRC and non-CA patients. No pronounced differences were found between the two groups but a lower tendency was seen in both Shannon and Chao1 indexes of preCA_CRC patients (Figures 4A,B). Analysis of beta diversity with PCoA showed parallel clusterings in two groups (Adonis test, *p* = 0.55, Figure 4C).

To identify differentially abundant taxa, we performed statistical analysis of taxonomic and functional profiles (STAMP), found that the abundance of bacterial microbiota was generally reduced in preCA_CRC patients. The abundance of some protective species, such as *Sutterella wadsworthensis* (anti-inflammation) (38) and *Flavonifractor plautii* (SCFAs biosynthesis) (39) further decreased in preCA_CRC patients, and the abundance of biomarker *M. funiformis* decreased as well (Figure 4D).

### Correlations Between the Progression of CRC and Bacterial Microbiota

**Figure 5:**
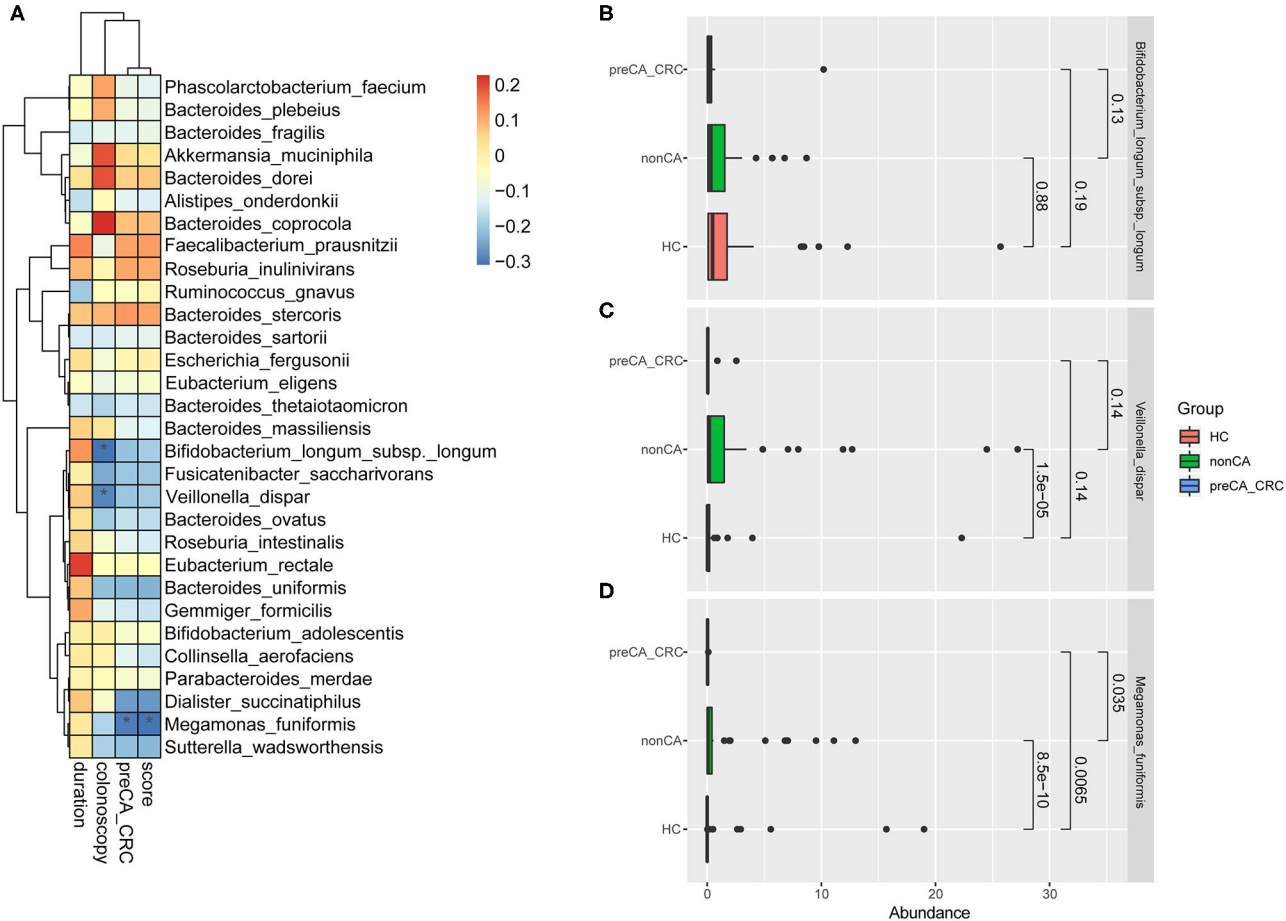
Correlation analysis of the progression of CRC and bacterial abundance. **(A)** Correlation analysis about bacterial microbiota and the progression of CRC based on duration after cholecystectomy, results of colonoscopy, complicating with preCA_CRC and the score of mucosal pathology. Spearman's correlation was performed. *p < 0.05. R 3.5.1 with the *pheatmap* package was used for data visualization. Abundance of **(B)** *Bifidobacterium longum* subsp. *longum*, **(C)** *Veillonella dispar* and **(D)** *Megamonas funiformis* in HC, non-CA and preCA_CRC groups, respectively. HC, healthy controls; non-CA, non-cancerous PC patients; preCA_CRC, PC patients with precancerous lesions and/or CRC.

In this study, we set up a new scoring method for colonoscopic biopsy and applied it as a factor of the progression of CRC (Table 2). With the increase of score, the cancer progressed to a later stage. Taking the duration after cholecystectomy, results of colonoscopy, complicating with preCA_CRC and the score of colonoscopic biopsy as variables of the progression of CRC into account, we found that the abundance of *M. funiformis, Veillonella dispar (V. dispar)*, and *Bifidobacterium longum* subsp. *longum (B. longum* subsp. *longum)* was significantly correlated with the progression of CRC after cholecystectomy (Figure 5A).

**Table 2:** A new scoring for mucosal pathology in post-cholecystectomy patients.

**Mucosal pathological results**	**Post-cholecystectomy**	**Score**
	**(*n* = 52)**	
No cancer sign	43 (82.7%)	0
Low-grade intraepithelial neoplasia	6 (11.5%)	1
High-grade intraepithelial neoplasia	1 (2.0%)	2
Invasive cancer	2 (3.8%)	3

Figures 5B–D detailed the abundant alterations of the above three species in HC, non-CA and preCA_CRC groups. The charts showed that the abundance of *M. funiformis* remarkably increased at first in non-CA group compared with healthy controls (*p* = 8.5e-10), and significantly decreased later in preCA_CRC group compared with non-CA group (*p* = 0.0065); additionally, *V. dispar* also had a semblable trend. In contrast, *B. longum* subsp. *longum* showed a continuous declination trend in abundance from HC to preCA_CRC group. Based on the above data, we inferred that bacterial dysbiosis might affected the progression of CRC after cholecystectomy.

## Discussion

In the past, the impact of cholecystectomy on health was insufficiently understood. However, more patients experienced gastrointestinal symptoms such as dyspepsia and diarrhea, which were difficult to relieve after cholecystectomy. Meanwhile, several meta-analysis suggested that the prevalence rate of CRC was increased (9–12). It was proved that gut microbiota was involved in the pathogenesis of many diseases such as CRC (13), inflammatory bowel disease (IBD) and irritable bowel syndrome (IBS) (40). To fully realize the potential of cholecystectomy, we need to make further careful studies on the alterations of gut microbiota in PC patients, which may provide new ideas for the study of related-diseases after cholecystectomy and new treatment strategies for PC patients.

Herein, we depicted the overall structure of bacterial microbiota by 16S rRNA gene sequencing, demonstrated that bacterial dysbiosis after cholecystectomy was characterized by distinct microbial composition and altered relative abundances in species with specific functions. Based on the data, two biomarkers were screened out for distinguishing PC patients from HC subjects, and the duration after cholecystectomy mainly affected bacterial composition. Note that bacterial dysbiosis after cholecystectomy was likely associated with CRC in PC patients.

Previous studies found that the circadian excretion rhythm of BAs disappeared after cholecystectomy, along with increases of bile acid synthesis and bile acid enterohepatic recirculation rate (24, 41–43). In our study, the bacterial species involved in bile acid metabolism had quite high abundance in PC patients, and it was probably ascribed to the enriched BAs exposure. Butyrate was the main energy source for colon cells and had protective effects against CRC and IBD (44, 45). Furthermore, a reduced abundance of protective bacteria which participated in SCFAs biosynthesis including butyrate (46–48), such as *F. prausnitzii* and *R. faecis*, were observed in our study, and it was surmised that decreased production of SCFAs by microbiota may affect carcinogenesis of CRC. Other than the characters above-mentioned, PC biomarker *L. mucosae* was reported to be interfered with by surfactant protein D (SP-D), which was synthesized and secreted by gallbladder in mice (49). Whether there were similar bacteria-protein interactions in the human gallbladder still needed to be verified.

Bacterial compositional state was shaped by many environmental factors but also potentially self-reinforcing. Duration after cholecystectomy was observed as a vital factor which affected bacterial composition in PC patients, and with the increase of duration after cholecystectomy, the bacterial composition changed more obviously. The external influence might trigger a compositional shift which gradually formed an adapted bacterial composition, and then bacterial composition possibly fed back to the host in multifarious ways, for example, via the production of certain metabolites like bile acids (50).

To investigate the role of bacterial microbiota in the pathogenesis and progression of CRC, we further analyzed the bacterial differences between the two subgroups of PC patients. preCA_CRC patients were enrolled after cholecystectomy at a mean duration of 11.17 (±10.34) years, and the mean duration was 9.13 (±7.55) years in non-CA patients. There was no significant difference in the mean duration after cholecystectomy (*t* = −0.690, *p* = 0.493), but a higher tendency was seen in preCA_CRC patients. In addition, basic clinical data, such as gender, clinical comorbidities, and laboratory tests, were not significantly different between preCA_CRC and non-CA groups. Furthermore, 66.7% of the preCA_CRC patients (*n* = 6) had mucosal lesions located in the right colon (Table 3), and these findings supported some conclusions in previous meta-analyses (9–12). Low bacterial diversity and reduced abundance of protective bacteria (mainly producing SCFAs) were considered to be major types of gut dysbiosis of CRC (51, 52); and it was widely reported that *Bacteroides fragilis, Fusobacterium nucleatum*, and *Prevotella intermedia* had increased in abundance of sporadic CRC patients (53, 54). Our findings were slightly different from bacterial alterations of sporadic CRC patients, and the reasons may lie in the small samples of preCA_CRC patients. We found the accumulation of most *Bacteroides* species, *Prevotella copri*, and *Fusobacterium varium* after cholecystectomy, and these species were from the same genera with the characteristic species of sporadic CRC. Some studies reported that the dominant species of *Bacteroides, Paraprevotella, Eubacterium* in fecal samples of CRC patients were often different (53). Additionally, the abundance of *M. funiformis, V. dispar*, and *B. longum subsp. longum* was correlated with the progression of CRC, which might play a pivotal role in related-disease studies. Based on these data, we speculated that CRC patients deduced from PC patients might have a distinctive bacterial profile when compared to sporadic CRC patients.

**Table 3:** The lesion distribution in preCA_CRC patients.

**Segment**	**preCA_CRC patients (*n* = 9)**
Ascending colon	5 (55.6%)
Right transverse colon	1 (11.1%)
Left transverse colon	0
Descending colon	1 (11.1%)
Sigmoid colon	2 (22.2%)
Rectum	0

Additionally, the guideline suggested that the general-risk population recommended will start screening colonoscopy at the age of 50 to find early CRC and precancerous lesions (55). According to previous studies and the results of the functional annotation of bacterial microbiota in our study, cholecystectomy may increase the risk of CRC in postoperative patients compared with healthy people. The age distribution during cholecystectomy is wide due to various causes such as acute cholecystitis, chronic cholecystitis, gallstones, and traumatic cholecyst rupture. Therefore, the results of our study suggested that regardless of age, patients after cholecystectomy were recommended to screen colonoscopy to detect early CRC and precancerous lesions.

Even their tentative conclusions were debatable, previous studies had partly reported the changes of bacterial microbiota after cholecystectomy by sequencing of fecal samples (17–19). Nevertheless, the impact of environmental factors on these bacterial alterations has not been analyzed, and the link between bacterial alterations and postoperative related-diseases has not received enough attention. The major advantages of our study contained subjects enrolled with colonoscopic biopsy, considerations of clinical characteristics and environmental factors, bacterial screening at the species level, and correlation analysis on bacterial alterations after cholecystectomy and CRC. However, our data provide evidence of association, not causality, and further studies are guaranteed to clarify how the disease-associated bacteria play a role in carcinogenesis and progression of CRC. The small sample size of preCA_CRC patients was indeed a limitation of our study. In the future, we plan to conduct a controlled study on patients with sporadic CRC and post-cholecystectomy patients with CRC to better explain the impact of cholecystectomy on the incidence of CRC.

In conclusion, our study showed specific bacterial alterations after cholecystectomy, and bacterial dysbiosis likely associated with carcinogenesis and progression of CRC in PC patients. Furthermore, duration after cholecystectomy notably affected bacterial composition in PC patients. Our findings provide novel insights into related-disease studies after cholecystectomy, and the long-term follow-up of PC patients should attract more attention. It reminds us of broad considerations when implementing cholecystectomy in clinic.

## Data Availability Statement

The datasets presented in this study can be found in online repositories. The names of the repository/repositories and accession number(s) can be found below: https://www.ncbi.nlm.nih.gov/, PRJNA541484.

## Ethics Statement

The studies involving human participants were reviewed and approved by the Institutional Medical Ethics Review Board of Peking University People's Hospital (Document No. 2018PHB035-01). The patients/participants provided their written informed consent to participate in this study.

## Author Contributions

YL, XR, and JX designed this study. XR, YuZ, GC, YiZ, and QH performed sample and clinical information collection. XR and JX performed data analysis and visualization. XR performed manuscript writing. YL and JX revised this article. All authors contributed to the article and approved the submitted version.

## Conflict of Interest

The authors declare that the research was conducted in the absence of any commercial or financial relationships that could be construed as a potential conflict of interest.

## Abbreviations

ALP: alkaline phosphatase
ALT: alanine aminotransferase
AST: aspartate aminotransferase
AUC: area under the curve
BAs: bile acids
BMI: body mass index
BSH: bile salt hydrolase
CHD: coronary atherosclerotic heart disease
CRC: colorectal cancer
GGT: glutamyl transpeptidase
HB: hemoglobin
HBP: hypertension
HC: healthy control
HLP: hyperlipemia
IBD: inflammatory bowel disease
IBS: irritable bowel syndrome
NAFLD: non-alcoholic fatty liver disease
NE%: neutrophilic granulocyte percentage
OTU: Operational Taxonomic Unit
PC: post-cholecystectomy
PCoA: principal coordinate analysis
PLT: blood platelet count
ROC: receiver operating characteristic curve
SCFAs: short-chain fatty acids
SP-D: surfactant protein D
STAMP: statistical analysis of taxonomic and functional Profiles
T2DM: type 2 diabetes mellitus
TBA: total bile acid
WBC: white blood cell count.
